# Cuts to Medicaid Adult Dental Benefits and Savings for US States

**DOI:** 10.1001/jamahealthforum.2026.3848

**Published:** 2026-10-16

**Authors:** Hawazin W. Elani, Ellen Meara

**Affiliations:** 1 Department of Oral Health Policy and Epidemiology, Harvard School of Dental Medicine, Boston, Massachusetts; 2 Department of Health Policy and Management, Harvard T.H. Chan School of Public Health, Boston, Massachusetts

## Abstract

This cross-sectional study explores how much US states might save by eliminating Medicaid adult dental benefits.

## Introduction

Medicaid adult dental benefits occupy an unusual role in US health policy. Although dental care prevents pain and infection, maintains oral function, and supports overall health,^1^ adult dental benefits remain optional for states and are frequently cut during fiscal downturns.^2^ After a decade in which states offering comprehensive benefits increased from 11 to 35,^2^ proposals to scale back coverage are re-emerging. The key policy question is how much states would actually save by eliminating them.

## Methods

We conducted a state-level analysis of Medicaid fee-for-service (FFS) dental spending from Medicaid Budget and Expenditure System data across all 50 states and Washington, DC, from 2013 to 2024.^3^ Adult dental benefit generosity was classified as none, emergency only, limited, or comprehensive.^2^ Spending was converted to 2024 US dollars. Enrollment-stratified analyses used adult and child FFS enrollment data available for 2016 through 2022. We calculated federal and state shares, including each state’s observed state percentage of FFS dental spending and per-enrollee spending. We used logistic regression with year fixed effects and state-clustered standard errors to examine the association between statutory Federal Medical Assistance Percentage (FMAP) and comprehensive adult dental coverage vs all other benefit levels (eMethods in Supplement 1). Statistical tests were 2 sided, with *P* < .05 considered statistically significant.

The Harvard Faculty of Medicine institutional review board determined that this study was not human participant research and waived need for review and informed consent. We followed the Strengthening the Reporting of Observational Studies in Epidemiology (STROBE) reporting guidelines.

## Results

From 2013 through 2024, the observed federal percentage of Medicaid FFS dental spending ranged from 50.4% to 79.4% (median, 66.1%). In 2024, the state-financed percentage of FFS dental spending—the fraction of any dental cut retained as state savings—was generally well below 50% (Figure 1); 16 states received at least 70% of FFS dental spending from federal funds, and 5 received more than 75%. Thus, reducing $1 in dental spending yields only about $0.20 to $0.50 in state savings, depending on matching rates; the remainder reflects forgone federal funds.

**Figure 1.**
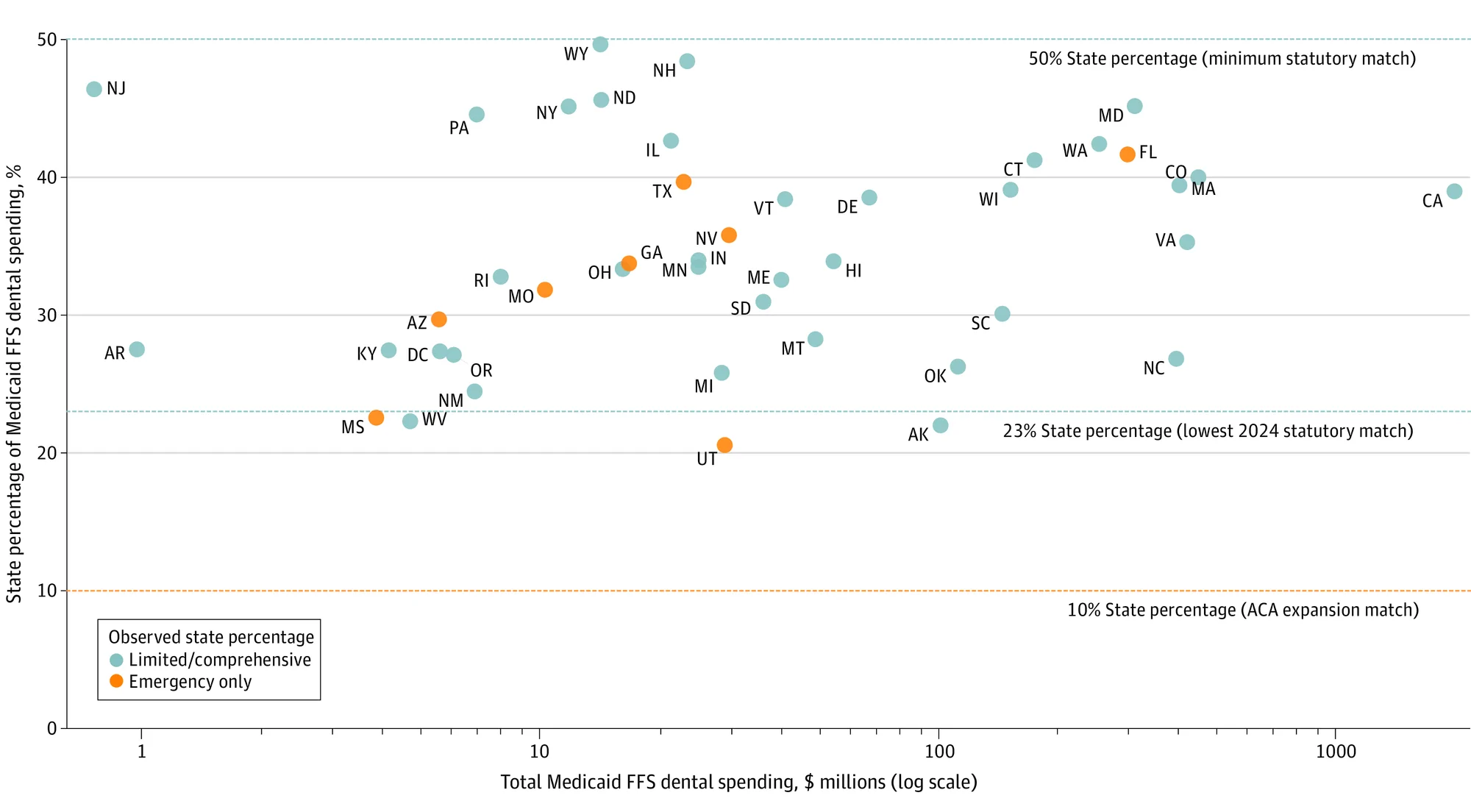
Dot Plot of Total Medicaid Fee-for-Service (FFS) Dental Spending and State Percentage of Total, 2024 Each point represents a US state or Washington, DC. The x-axis shows 2024 Medicaid FFS dental spending, combining adults and children, in millions of 2024 US dollars. The y-axis shows the observed (average) state percentage of total Medicaid FFS dental spending, interpreted as the maximum approximate state-budget savings per dollar of reduced FFS dental spending; the remainder represents forgone federal funds. Because Medicaid Budget and Expenditure System/Children’s Health Insurance Program Budget and Expenditure System reports total FFS dental spending, these are average, not marginal, savings. In Medicaid expansion states, the observed state percentage blends the higher state percentage for traditional enrollees (100% minus the statutory Federal Medical Assistance Percentage) with the 10% state share for adults with Affordable Care Act (ACA) expansion matched at 90%. Thus, cuts in dental spending among adults with expansion benefits save states 10% per dollar spending cut, while cuts in dental spending among enrollees with traditional Medicaid benefits save more (up to 50% per dollar spent, since 50% is the lowest possible Federal Medical Assistance Percentage). Point colors denote adult Medicaid FFS dental benefit level. States with no adult dental benefit in FFS are excluded. Idaho, Nebraska, and Kansas are also excluded because adult dental care is delivered primarily through capitated managed care in those states, and reported FFS dental spending was negligible. Spending excludes Children’s Health Insurance Program dental spending and dental spending embedded in managed care capitation.

National Medicaid FFS dental spending increased from about $4.8 billion in 2013 to more than $6 billion in 2024 but remained below 1% of total FFS Medicaid spending. Average dental spending increased from $168 per enrollee in 2016 to $203 in 2022 (in 2024 US dollars). In 2022, children’s per-enrollee spending averaged $662 across FFS Medicaid and the Children’s Health Insurance Program (CHIP) vs $171 for adults (Figure 2). Adults, however, accounted for 27.7 million FFS enrollees vs 1.9 million children, or approximately 79% of combined dental spending.

**Figure 2.**
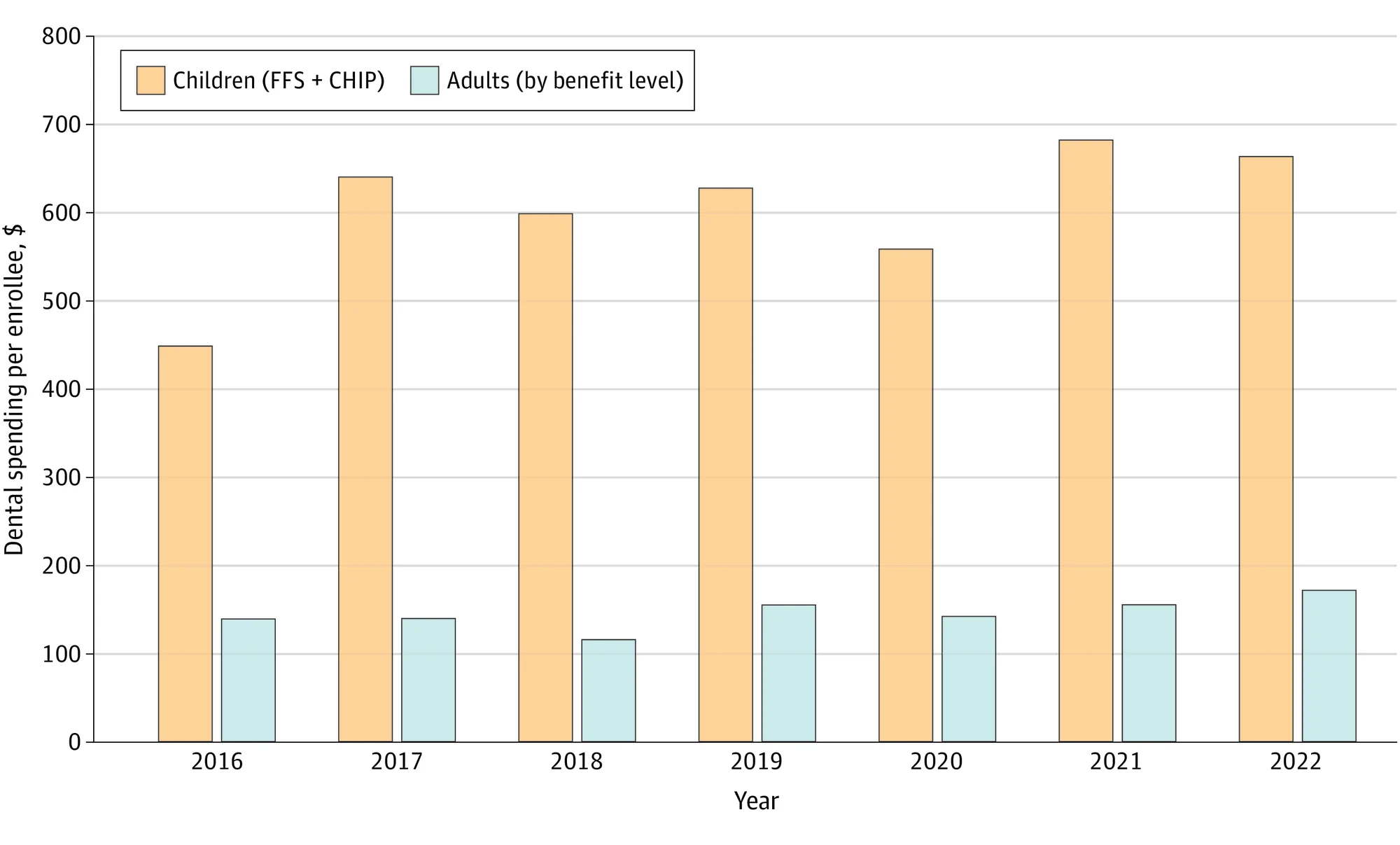
Bar Chart of per-Enrollee Dental Spending for Children and Adults in Medicaid and the Children’s Health Insurance Program, 2016-2022 Children’s per-enrollee spending includes fee-for-service (FFS) Medicaid dental services covered under the Early and Periodic Screening, Diagnostic, and Treatment benefit and the Children’s Health Insurance Program (CHIP) dental spending. Per-enrollee calculations use FFS enrollment data by age, available through 2022. Adult per-enrollee spending is calculated from FFS Medicaid dental expenditures and weighted by each state’s adult dental benefit level utilization rates from the American Dental Association (eMethods in Supplement 1). All values are expressed in 2024 US dollars.

West Virginia illustrates the fiscal distinction. In 2024, its Medicaid program spent $4.95 million on FFS dental services, 77.9% federally financed. Thus, eliminating adult dental benefits would reduce total expenditures by roughly $4.1 million but save the state only $0.91 million while forgoing $3.2 million in federal funds, or about $3.50 in federal dollars for every $1 in state savings.

From 2013 through 2024, a 10–percentage point increase in statutory FMAP was associated with a 14–percentage point lower probability that a state offered comprehensive adult dental benefits vs all other benefit levels (*P* = .02). In 2024, 11 states with an FMAP at or above 60% offered emergency-only or no adult dental coverage, including Mississippi and Alabama, where FMAPs were at or above 73%.

## Discussion

In this cross-sectional study, eliminating adult dental benefits showed reduction in total Medicaid dental expenditures, but those reductions should not be interpreted as state-budget savings. Because federal dollars finance most Medicaid FFS dental spending, states retain only a fraction of apparent savings. The federal share is also elevated by pediatric dental services, mandatory under the Early and Periodic Screening, Diagnostic, and Treatment benefit,^4^ and by CHIP spending, which receives an enhanced federal matching rate roughly 15 percentage points above traditional FMAP.^5^ In many states, roughly two-thirds of FFS dental spending is federally financed, meaning most reduced spending would otherwise have been paid federally. Benefit design therefore reflects fiscal incentives alongside administrative and political factors, as several high-FMAP states offer minimal adult coverage.

Benefit reductions may shift costs to emergency departments and medical care. In 2019, Medicaid was the expected payer for approximately 41% of treat-and-release emergency department visits for nontraumatic dental conditions and 38% of associated costs.^6^ Untreated oral disease may worsen chronic disease management, although evidence on medical spending offsets remains mixed.^1^ An American Dental Association analysis estimated that eliminating adult Medicaid dental benefits could increase US health care costs by approximately $1.9 billion through greater emergency department use and unmet periodontal treatment needs.^7^ Episodic dental care may expose patients to antibiotics or analgesics without definitive treatment,^8^ including inappropriate opioid prescribing and repeat utilization.

These estimates have limitations. The Medicaid Budget and Expenditure System/CHIP Budget and Expenditure System does not isolate dental spending embedded in managed care capitation, where most adult Medicaid dental services are delivered. It also combines adult and pediatric dental spending, limiting state-specific savings estimates. As a result, these data understate total Medicaid dental spending. More accurate fiscal assessments would require separate reporting of dental expenditures by managed care organizations and federal reporting that distinguishes adult from pediatric dental spending.

## Notes

Supplement 1.
eMethods

eReferences

Supplement 2.
Data Sharing Statement
