# Supplementary material for: Cuts to Medicaid Adult Dental Benefits and Savings for US States
Source: JAMA Health Forum. 2026 Oct 16;7(10):e263848. doi: 10.1001/jamahealthforum.2026.3848 (PMC13675816; doi:10.1001/jamahealthforum.2026.3848)
Supplement: Supplementary file 1 [file jamahealthforum-e263848-s001.pdf]

Supplemental Online Content

Elani HW, Meara E. Cuts to Medicaid adult dental benefits and savings for US states. *JAMA Health Forum*. 2026;7(10):e263848. doi:10.1001/jamahealthforum.2026.3848

eMethods
eReferences

This supplemental material has been provided by the authors to give readers additional information about their work.

© 2026 Elani HW and Meara E. *JAMA Health Forum*.

eMethods.

I. Data sources, variables, and study sample

MBES/CBES. We conducted a state-level descriptive analysis of Medicaid dental spending using the Medicaid Budget and Expenditure System/CMS-64 (MBES/CBES) fee-for-service (FFS) expenditure files for 2013 through 2024 (50 states and the District of Columbia).[1] For each state-year and service category, MBES/CBES reports total computable expenditures and their financing components, including the federal share and state share. Our primary spending measure was FFS dental spending, defined using MBES/CBES service category records labeled "Dental Services."

Separating Medicaid from CHIP. Because MBES/CBES includes both Medicaid and Children's Health Insurance Program (CHIP) spending, we distinguished Medicaid from CHIP service categories. We treated CHIP dental spending (service category labeled "C-Dental Services") as separate from Medicaid dental spending to avoid conflating Medicaid financing with CHIP's enhanced matching. CHIP spending was included only in analyses limited to child per-enrollee analyses because our enrollment source does not distinguish Medicaid from CHIP child enrollees (and therefore does not support a Medicaid-only child denominator). Otherwise, all results refer to Medicaid FFS dental spending.

Adult Medicaid dental benefits. Adult Medicaid dental benefit generosity was measured using a state-year database constructed by our team from several data sources.[2] Adult dental benefits were classified into four categories: none, emergency-only, limited, and comprehensive. Benefits were classified as none (no adult dental services), emergency-only (coverage limited to treatment of acute pain, infection, or trauma), limited (fewer than 100 diagnostic, preventive, and minor restorative services, with an annual cap ≤$1,000), or comprehensive (>100 diagnostic, preventive, and restorative services, with an annual cap ≥$1,000).[3]

Enrollment. We used state-year enrollment counts stratified by (a) FFS versus managed care and (b) adults versus children for 2016–2022. Age-stratified national FFS enrollment was not available beyond 2022 in our enrollment source. In addition, FFS child counts in our enrollment source are not separable into Medicaid versus CHIP children. To construct enrollment denominators for per-enrollee analyses, we relied on CMS Transformed Medicaid Statistical Information System monthly eligibility and enrollment data and supplementary CMS sources on delivery system (FFS vs managed care) to produce state-year estimates of adult and child enrollment in FFS versus managed care.[4]

Inflation adjustment. All spending amounts reported in the manuscript were converted to 2024 dollars using year-specific inflation factors and are presented as inflation-adjusted totals.[5]

Statutory FMAP. Statutory Federal Medical Assistance Percentage (FMAP) values by state and year were obtained from the Kaiser Family Foundation's compilation of federal matching rates, which are derived from federal statute and CMS guidance.[6] These values were used to characterize cross-state variation in baseline federal matching rates and to examine the relationship between statutory FMAP and adult dental benefit generosity.

Managed care limitation. MBES/CBES does not isolate dental spending paid through capitation (e.g., dental services embedded in MCO capitation or paid through separate capitated dental arrangements). Accordingly, MBES/CBES-based dental expenditures should be interpreted as FFS-dental spending, and not as complete Medicaid dental spending in states where dental care is primarily delivered through managed care.

© 2026 Elani HW and Meara E. *JAMA Health Forum*.

II. National and state financing measures and trend estimates

For national trend estimates, we aggregated MBES/CBES FFS dental spending to the national level by summing state-year expenditure amounts across all 50 states and the District of Columbia. We reported (1) total national Medicaid FFS dental spending by year, (2) dental spending as a percentage of total Medicaid FFS spending, and (3) trends in federal versus state components of FFS dental spending over time, calculated from MBES/CBES federal and state expenditure fields.

For cross-sectional comparisons of Medicaid dental financing in 2024, we calculated each state's observed federal share of Medicaid FFS dental spending as:

$$Observed\ Federal\ Percentage_{s,2024} = \frac{Federal\ FFS\ Dental\ Spending_{s,2024}}{Total\ FFS\ Dental\ Spending_{s,2024}}\ x\ 100 \qquad \text{(Equation 1)}$$

We used the observed state percentage to approximate the maximum state-budget savings per dollar of reduced Medicaid FFS dental spending. For example, an observed state percentage of 30% implies that a $1 reduction in FFS dental spending would translate into, at most, approximately $0.30 in state-budget savings, while the remaining $0.70 would represent forgone federal funds. This estimate should be interpreted as an average financing percentage, not an observed or marginal savings estimate for a specific adult dental benefit cut. In Medicaid expansion states, the observed state percentage blends the 10% state share for ACA expansion adults matched at 90% federal funding with the higher state share for traditional Medicaid eligibility groups. Therefore, actual state savings from adult dental benefit reductions could be lower if cuts disproportionately affect expansion adults, or somewhat higher if cuts disproportionately affect traditional Medicaid enrollees.

Total Medicaid FFS dental spending was defined as the MBES/CBES Total Computable amount for the Dental Services category, which represents the sum of federal and state allowable expenditures. The observed federal percentage therefore reflects the combined effects of statutory FMAP, enhanced federal matching for Affordable Care Act expansion adults, and other applicable federal matching provisions embedded in CMS expenditure reporting.

III. Per-enrollee spending, adult-versus-child comparisons

To account for differences in population size, we estimated FFS dental spending per enrollee by dividing state-year Medicaid FFS dental spending by the corresponding enrollment denominator. Enrollment by age group was available through 2022; therefore, all analyses that required separate adult and child denominators use 2022 as the most recent year with complete age-stratified FFS enrollment. Per-enrollee estimates were calculated using FFS spending only, because MBES/CBES does not permit extraction of dental spending embedded in capitation payments.

Allocation method (utilization-weighted). MBES/CBES does not report FFS dental spending separately for adults versus children. To estimate adult-versus-child per-enrollee amounts, we allocated each state-year's total Medicaid FFS dental spending between adults and children using benefit-specific dental utilization rates for adults and treating children as having comprehensive coverage because pediatric dental is mandatory under EPSDT. We used adult utilization rates by benefit category (none, emergency-only,

© 2026 Elani HW and Meara E. *JAMA Health Forum*.

limited, comprehensive) from estimates reported by the American Dental Association Health Policy Institute.[7]

For each state-year, we computed:

- *Expected adult users = FFS adult enrollment × adult utilization rate (based on the state's adult benefit level), and*
- *Expected child users = FFS child enrollment × child utilization rate (EPSDT/comprehensive).*

We then allocated each state-year's Medicaid FFS dental spending proportionally to expected users and calculated adult and child per-enrollee spending as:

$$Adult\ per\ enrollee_{s,t} = \frac{FFS\ dental\ spending_{s,t} \times adult\ expected\text{-}user\ share_{s,t}}{FFS\ adult\ enrollment_{s,t}} \qquad \text{(Equation 2)}$$

$$Child\ per\ enrollee_{s,t} = \frac{FFS\ dental\ spending_{s,t} \times child\ expected\text{-}user\ share_{s,t} + CHIP\ dental\ spending^{\dagger}_{s,t}}{FFS\ child\ enrollment_{s,t}} \qquad \text{(Equation 3)}$$

† CHIP dental spending was included only when explicitly indicated (limited to child per-enrollee analyses) because FFS child enrollment in our enrollment source cannot be separated into Medicaid versus CHIP children. When CHIP spending was included, we retained the same combined child denominator.

IV. Statistical model: statutory FMAP and comprehensive adult dental benefits

We examined whether baseline federal matching rates were associated with comprehensive adult Medicaid dental benefits using a state-year panel binary logistic regression model for 2013–2024 (50 states and DC). The outcome was comprehensive adult dental benefits versus all other coverage levels. The main predictor was statutory FMAP in percentage points. The model included year fixed effects to account for national time trends, and standard errors were clustered by state. To present results on an interpretable scale, we reported the change in predicted probability associated with a 10–percentage-point increase in statutory FMAP.

V. Illustrative single-state example

To illustrate the fiscal distinction between total expenditure reductions and state-budget savings, we present a worked single-state example. Because MBES/CBES reports total FFS dental spending but does not separate adult from pediatric dental spending, we estimated adult dental spending by applying the national adult share of FFS dental spending to the state's total FFS dental expenditure. We then applied the state's observed federal and state percentages of FFS dental spending to estimate the portion of any reduction that would accrue as state-budget savings versus forgone federal funds. Because the federal-to-state savings ratio depends only on the observed state percentage, it is unaffected by the adult-share assumption; only the estimated dollar magnitudes depend on the national adult share.

© 2026 Elani HW and Meara E. *JAMA Health Forum*.

eReferences

1. Centers for Medicare & Medicaid Services. Medicaid and CHIP Budget and Expenditure System (MBES/CBES). 2025. Accessed January 15,2026. https://www.medicaid.gov/medicaid/financial-management/state-expenditure-reporting-for-medicaid-chip/expenditure-reports-mbescbes
2. Elani H, Benjamin DS, Ichiro K, Renuka T, Meredith R. Database of State-Level Variation in Medicaid Adult Dental Coverage. Version 1. Harvard Dataverse; 2026. Accessed January 15, 2026. https://doi.org/10.7910/DVN/LPHSLV
3. Centers for Medicare & Medicaid Services. Medicaid & CHIP Beneficiaries at a Glance: Oral Health. 2023. Accessed January 15, 2026. https://www.medicaid.gov/medicaid/benefits/downloads/2023-oral-health-at-a-glance.pdf
4. Medicaid.gov. Major eligibility group information for Medicaid and CHIP beneficiaries by month. 2024. January 15, 2026. https://data.medicaid.gov/dataset/ea9b7db3-db71-4663-b4e1-67e11d1d4fcc
5. US Bureau of Labor Statistics. Consumer Price Index. 2023. Accessed January 15, 2026. https://www.bls.gov/cpi/tables/supplemental-files/
6. Henry J. Kaiser Family Foundation. Federal Medical Assistance Percentage (FMAP) for Medicaid and multiplier. 2024. Accessed January 15, 2026. https://www.kff.org/medicaid/state-indicator/federal-matching-rate-and-multiplier/?currentTimeframe=3&sortModel=%7B%22colId%22:%22Location%22,%22sort%22:%22asc%22%7D
7. Vujicic M, Fosse C, Reusch C, Burroughs M. Making the Case for Dental Coverage for Adults in All State Medicaid Programs. American Dental Association Health Policy Institute; 2021. Accessed January 15, 2026. https://www.ada.org/~/media/ADA/Science%20and%20Research/HPI/Files/WhitePaper_0721.pdf

© 2026 Elani HW and Meara E. *JAMA Health Forum*.
