# Supplementary material for: Cuts to Medicaid Adult Dental Benefits and Savings for US States
Source: JAMA Health Forum. 2026 Oct 16;7(10):e263848. doi: 10.1001/jamahealthforum.2026.3848 (PMC13675816; doi:10.1001/jamahealthforum.2026.3848)
Supplement: Supplementary file 2 [file jamahealthforum-e263848-s002.pdf]

Data Sharing Statement

Elani. Cuts to Medicaid Adult Dental Benefits and Savings for US States. *JAMA Health Forum*. Published October 16, 2026. doi:10.1001/jamahealthforum.2026.3848

Data
Data available: No

Additional Information
Explanation for why data not available: Data is publicly available
